# Supplementary material for: Evolution and Applications of Recent Sensing Technology for Occupational Risk Assessment: A Rapid Review of the Literature
Source: Sensors (Basel). 2022 Jun 27;22(13):4841. doi: 10.3390/s22134841 (PMC9269318; doi:10.3390/s22134841)

sensors

MDPI

Review

Supplementary Materials: Evolution and applications of recent sensing technology for occupational risk assessment: a rapid review of the literature

Giacomo Fanti, Andrea Spinazzè, Francesca Borghi, Sabrina Rovelli, Davide Campagnolo, Marta Keller, Andrea Borghi, Andrea Cattaneo, Emanuele Cauda and Domenico Maria Cavallo

Table S1. Studies object of the review. In this table are reported all the sources considered in this review, divided by author, title, reference and the corresponding number of citations in the text.

First Author	Title	Journal	Year of publication	Citation
G. Atzeni	A 1.01 NEF Low-Noise Amplifier Using Complementary Parametric Amplification	IEEE Transactions on Circuits and Systems	2021	[60]
S. Lim	A narrative review on contemporary and emerging uses of inertial sensing in occupational ergonomics	International Journal of Industrial Ergonomics	2020	[71]
K. Isiugo	Assessing the accuracy of commercially available gas sensors for the measurement of ambient ozone and nitrogen dioxide	Journal of Occupational and Environmental Hygiene	2018	[26]
P.H. Dalton	Chemosensory loss: Functional consequences of the world trade center disaster	Environmental Health Perspective	2010	[55]
B. Shamasunder	Community-based health and exposure study around urban oil developments in South Los Angeles	International Journal of Environmental Reasearch and Public Health MDPI	2018	[52]
L. Salo	Concentrations and size distributions of particle lung-deposited surface area (LDSA) in an underground mine	Aerosol and Air Quality Research	2021	[44]
O.R. Ogunseiju	Digital twin-driven framework for improving self-management of ergonomic risks	Smart and Sustainable Built Environment	2021	[43]
J. Sabburg	Effect of cloud on UVA and exposure to humans	Photochemistry and Photobiology	2001	[37]
L.N. Johannessen	Embedded systems and the Internet of Things: Can low-cost gas sensors be used in risk assessment of occupational exposure?	Journal of Occupational and Environmental Hygiene	2020	[38]
N.D. Nath	Ergonomic analysis of construction worker's body postures using wearable mobile sensors	Applied Ergonomics	2017	[24]
M.M. Sugg	Estimating personal ambient temperature in moderately cold environments for occupationally exposed populations	Environmental Research	2019	[53]
C. Zuidema	Estimating personal exposures from a multi-hazard sensor network	Journal of Exposure Science & Environmental Epidemiology	2020	[11]
M. Le Feber	Ethics and privacy considerations before deploying sensor technologies for exposure assessment in the workplace: Results of a structured discussion amongst dutch	Annals of Work Exposures and Health	2021	[28]

J.M. Olegario	Evaluation of low-cost optical particle counters for agricultural exposure measurements	American Society of Agricultural and Biological Engineers	2021	[54]
G. Fanti	Features and practicability of the next-generation sensors and monitors for exposure assessment to airborne pollutants: A systematic review	Sensors - MDPI	2021	[8]
H. Goede	Future Prospects of occupational Exposure Modelling of Substances in the Context of Time-Resolved Sensor Data	Annals of Work Exposures and Health	2021	[14]
H.O. Austad	Hand-arm vibration exposure monitoring with wearable sensor module	pHealth 2013/ Studies in Health Technology and Informatic	2013	[56]
S. Sousan	Laboratory evaluation of low-cost optical particle counters for environmental and occupational exposures	Sensors - MDPI	2021	[31]
C. Zuidema	Mapping Occupational Hazards with a Multi-sensor Network in a Heavy-Vehicle Manufacturing Facility	Annals of Work Exposure and Health	2019	[45]
K.A. Baczynska	Measurements of UV-A exposure of commercial pilots using genesis-UV dosimeters	Atmophere MDPI	2020	[65]
H. Wan	Miniaturized planar room temperature ionic liquid electrochemical gas sensor for rapid multiple gas pollutants monitoring	Sensors and Actuators, B	2018	[57]
P. Zradziński	Modelling the influence of electromagnetic field on the user of a wearable iot device used in a WSN for monitoring and reducing hazards in the work environment	Sensors - MDPI	2020	[39]
C.J. Patel	Opportunities and challenges for environmental exposure assessment in population-based studies	American Association for Cancer Research AACR	2017	[27]
J.D. Berman	Optimizing a sensor network with data from hazard mapping demonstrated in a heavy-vehicle manufacturing facility	Annals of Work Exposure and Health	2018	[46]
A. Coca	Physiological monitoring in firefighter ensembles: Wearable plethysmographic sensor vest versus standard equipment	Journal of Occupational and Environmental Hygiene	2009	[36]
J. Taborri	Preventing and monitoring work-related diseases in firefighters: A literature review on sensor-based systems and future perspectives in robotic devices	International Journal of Environmental Research and Public Health	2021	[5]
B.W. Jo	Proximity warning and excavator control system for prevention of collision accidents	Sustainability	2017	[50]
T. Puester	Qualification of technical safety measures for the safe use of hand-held laser processing devices	Journal of laser applications	2018	[51]
R. Leghrib	Quantitative trace analysis of benzene using an array of plasma-treated metal-decorated carbon nanotubes and fuzzy adaptive resonant theory techniques	Analytica Chimica Acta	2011	[58]
J.D. Pleil	Rationale for developing tunable laser spectroscopy (TLS) technology for high resolution real-time carbon dioxide monitoring (capnography) in human breath	Journal of Breath Research	2021	[48]
Y. Shao	Real-time air monitoring of occupational exposures to particulate matter among hairdressers in Maryland: A pilot study	INDOORAIR	2021	[47]
K.B. Nelson	Reporting back environmental health data among outdoor occupational workers in the cold season in north carolina, usa	Southeastern Geographer	2020	[19]
A. Misistia	Sensor orientation and other factors which increase the blast overpressure reporting errors	PLOS ONE	2020	[40]
C. Zuidema	Sources of error and variability in particulate matter sensor network measurements	Journal of Occupational and Environmental Hygiene	2019	[41]

T. Puester	Technical safety measures for the safe use of hand-held laser processing devices	Journal of Laser Applications	2012	[18]
M.M. Sugg	Temporal and spatial variation in personal ambient temperatures for outdoor working populations in the southeastern USA	International Journal of Biometeorology	2018	[25]
M. Porta	Use of wearable sensors to assess patterns of trunk flexion in young and old workers in the Metalworking Industry	Ergonomics	2021	[49]
R. Alberto	Wearable monitoring devices for biomechanical risk assessment at work: Current status and future challenges - A systematic review	International Journal of Environmental Research and Public Health MDPI	2018	[42]
M.J. Buller	Wearable physiological monitoring for human thermal-work strain optimization	Journal of Applied Physiology	2018	[63]
E. Pievanelli	Wireless technology for occupational dosimetry implementing medium range sensor networks	International Symposium on Electromagnetic Theory	2013	[59]

Figure S1. Flowchart of the papers which are the object of this review (modified from Moher et al., 2009).

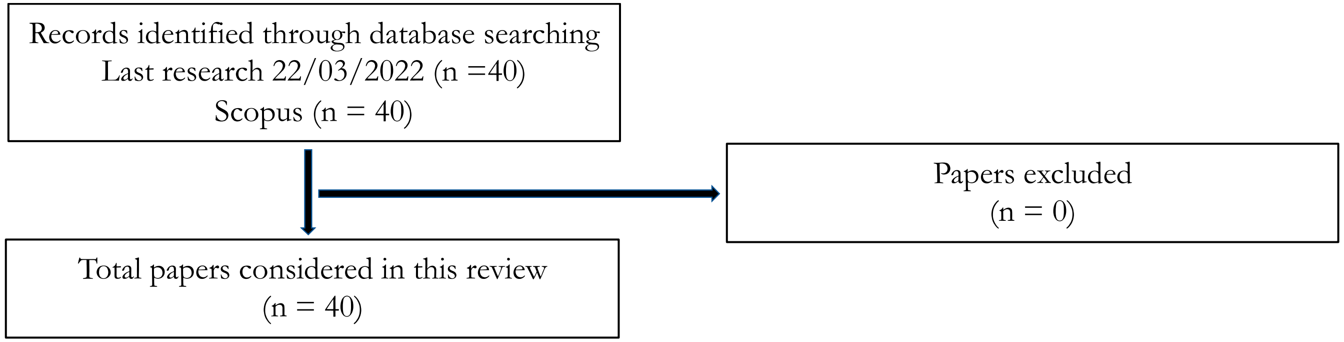

Supplement: Supplementary file 1 [file sensors-22-04841-s001.zip › sensors-1732110-supplementary.pdf]
